# Supplementary material for: Changes in emotional distress among Ontario education workers during the COVID-19 pandemic: 2021–2023
Source: PLoS One. 2025 Aug 18;20(8):e0330442. doi: 10.1371/journal.pone.0330442 (PMC12360544; doi:10.1371/journal.pone.0330442)
Supplement: S1 Table — (PDF) [file pone.0330442.s001.pdf]

S1 Table: Percent change in K10 scores per four weeks, Ontario education workers participating in the K10 sub-study *with five or more submissions*, February 18, 2021 to December 22, 2023.
Linear mixed effects model

**Variable**	**Percent change in K10 scores (95% CI)**
Time, per 4 weeks	**-0.5 (-0.6, -0.4)**
Mitigation level: Low<br>High	Referent<br>NA
Season: Winter<br>Autumn<br>Spring<br>Summer	Referent<br>**-1.7 (-3.1, -0.3)**<br>**-1.8 (-3.1, -0.4)**<br>**-13.9 (-15.0, -12.9)**
Return-to-workplace period: No<br>Yes	Referent<br>**3.9 (1.8, 6.0)**
Age, in years	**-0.5 (-0.7, -0.4)**
Gender: Female<br>Male	Referent<br>**-6.1 (-10.1, -2.1)**
People per bedroom	NA
Anti-anxiety, antidepression or anti-insomnia medication: No<br>Yes	Referent<br>**12.7 (8.2, 17.5)**
Subjective health: Poor/fair/good<br>Very good/excellent	Referent<br>**-14.4 (-17.1, -11.6)**
COVID-19 vaccine in last six months: None<br>One or more	Referent<br>**1.2 (0.1, 2.2)**
COVID-19 infection in last six months: None<br>One or more	Referent<br>NA
Postal district: Central Ontario<br>Eastern Ontario<br>Metropolitan Toronto<br>Northern Ontario<br>Southwestern Ontario	NA[1]
Occupation: Teacher/instructor<br>Administrative/clerical[2]<br>Educational assistant/ECE<br>Other, operational support[3]<br>Principal/VP<br>Other, professional support[4]	Referent<br>NA
Highest level of student contact: None<br>In room, often <2m<br>In room, rarely <2m<br>Physical contact	Referent<br>NA
Highest level of coworker contact: None<br>In room, often <2m<br>In room, rarely <2m<br>Physical contact	Referent<br>NA
Number of student contacts per week	NA

Bold: group significantly different ($p<0.05$) from referent group
ECE: early childhood educator; NA: not applicable (not included in model due to lack of significance)
[1] Sample size in some postal districts was too small to allow for variable inclusion in the sensitivity analysis
[2] Office and clerical staff, superintendent, human resources, finance, planner

[3] Information technology, audio-visual or computer technicians, bus driver, custodian, building maintenance (carpenter, plumber), cafeteria staff, lunchroom assistant
[4] Psychologist, social worker, therapist, librarian, registered nurse
