# Supplementary material for: Changes in emotional distress among Ontario education workers during the COVID-19 pandemic: 2021–2023
Source: PLoS One. 2025 Aug 18;20(8):e0330442. doi: 10.1371/journal.pone.0330442 (PMC12360544; doi:10.1371/journal.pone.0330442)
Supplement: Attachment — Submitted filename: Response to Reviewers_2025_02_06.docx [file pone.0330442.s003.docx]

February 6, 2025

**RE**: PONE-D-24-47690

Changes in emotional distress among Ontario education workers during the COVID-19 pandemic, PLOS ONE submission

**Response to Reviewers**

We thank the reviewers for their comprehensive reviews. Our responses are captured under each comment. Please note that when lines are referenced, we are referring to the manuscript that highlights changes to the original version, the file labelled “Revised Manuscript with Track Changes”.

**Editor comments**

*1. Please ensure that your manuscript meets PLOS ONE's style requirements, including those for file naming*.

We have reviewed this submission and ensured that we are compliant with the PLOS ONE style templates.

*2. We note that you have indicated that there are restrictions to data sharing for this study. Before we proceed with your manuscript, please address the following prompts:*

*If there are ethical or legal restrictions on sharing a de-identified data set, please explain them in detail (e.g., data contain potentially identifying or sensitive patient information, data are owned by a third-party organization, etc.) and who has imposed them (e.g., a Research Ethics Committee or Institutional Review Board, etc.). Please also provide contact information for a data access committee, ethics committee, or other institutional body to which data requests may be sent.*

The following was stated in the original submission:

The datasets generated and/or analysed during the current study are not publicly available due to information that could compromise the privacy of research participants but are available from the corresponding author on reasonable request.

This has been replaced with:

The datasets generated and/or analysed during the current study are not publicly available as the data file contains potentially identifying patient information. Requests for data access should be sent to:

Dr. Brenda L. Coleman, PhD

Clinical Scientist, Sinai Health, Toronto

Assistant Professor, Dalla Lana School of Public Health, University of Toronto

b.coleman@utoronto.ca

*3. PLOS requires an ORCID iD for the corresponding author in Editorial Manager on papers submitted after December 6th, 2016. Please ensure that you have an ORCID iD and that it is validated in Editorial Manager*.

The following information has been added: Dr. Brenda Coleman: 0000-0002-7144-4827

*4. Please review your reference list to ensure that it is complete and correct.*

The reference list has been reviewed to ensure compliance with PLOS ONE submission requirements.

**Additional Editor Comments:**

*1. Consider providing more detailed background information about the existing literature. For example, how does this study build upon prior research on the psychological impacts of COVID-19 on educators or other frontline workers?*

Additional information from longitudinal studies has been added (see lines 84-99).

*2. Clarify the rationale for focusing specifically on Ontario education workers. Is there something unique about this population that warrants investigation?*

Additional information regarding the importance of this group of essential workers has been added to the introduction (see lines 59-65). In addition, details regarding the generalizability of this information have also been added to the limitations section (see lines 379-381).

*3. Provide a clear statement of the research question or hypothesis.*

The study hypothesis is now stated (see lines 105-109).

*4. The inclusion criteria mention education workers aged 18-77 who were employed in Ontario schools and working an average of ≥8 hours/week, but the exclusion criteria for those who selected "neither male nor female" as their gender are vague. Clarify why participants who selected "neither male nor female" were excluded due to "small sample size." Providing more context on this decision, such as whether the data from non-binary participants was insufficient to draw meaningful conclusions, would make this clearer. Additionally, consider expanding on the inclusion and exclusion criteria in more detail for transparency, particularly if gender diversity is a relevant aspect to the study.*

More detail has now been provided regarding the decision to drop those who did not select male or female as their gender (see lines 123-126).

*5. The inclusion of COVID-19-specific factors (e.g., infection history, vaccination status) is mentioned, but their relationship to K10 scores is not fully explored or explained in the methodology.*

More details regarding how variables were created are now provided in the section “Survey instruments” (see lines 139-173).

*6. Report any statistical significance (e.g., p-values or confidence intervals) for the findings related to K10 scores and associated factors. For example, was the decrease in K10 scores over time statistically significant?*

The relationship between K10 scores and the four temporal variables was statistically significant (see lines 234-240). Other significant factors are described in lines 249-254. Further, as indicated in the notes for Table 2, all factors significantly associated with K10 distress scores are bolded.

*7. The relationship between specific periods (e.g., “return-to-workplace periods”) and distress could be elaborated further. How much variation in K10 scores was explained by these factors?*

Details of the relationship between the temporal variables and distress are provided in lines 240-246. The model conditional R^2^ and the marginal R^2^ are reported in the Table 2 notes and now also on lines 254-257.

*8. While revising your submission, please upload your figure files to the Preflight Analysis and Conversion Engine (PACE) digital diagnostic tool.*

The single figure that accompanies this paper has be uploaded to the PACE digital diagnostic tool.

**Reviewer comments**

*1. Is the manuscript technically sound, and do the data support the conclusions?*

*Reviewer #1: Yes*

*________________________________________*

*2. Has the statistical analysis been performed appropriately and rigorously?*

*Reviewer #1: Yes*

*________________________________________*

*3. Have the authors made all data underlying the findings in their manuscript fully available?*

*Reviewer #1: Yes*

*________________________________________*

*4. Is the manuscript presented in an intelligible fashion and written in standard English?*

*Reviewer #1: Yes*

________________________________________

**5. Review Comments to the Author**

Reviewer #1: I enjoyed reading your article and found the methods generally rigorous and the findings interesting. There were a few places where I wished for some clarification to understand what was done and how better:

*1) Is there a particular reason why you chose these specific start and end-dates; perhaps especially the end-dates?*

The study was started once funding was obtained. Initially, funding was requested for 14 months. Then, we applied for and received additional funding to continue the study until the end of 2023. New funding was not requested for the 2024 calendar year (see lines 115-122).

*2) I don't understand the mitigation scoring and variables. Would it be possible to explain what it is based on more clearly?*

Additional information has now been provided (see lines 168-173)

*3) With respect to the four-week intervals respecting the K-10 scores, I think I understand what you did based on the elaboration in the results, but here too I think the methodology could be described more clearly.*

We hope the new wording is clearer (see lines 157-158).

________________________________________

*6. PLOS authors have the option to publish the peer review history of their article. Do you want your identity to be public for this peer review? For information about this choice, including consent withdrawal, please see our Privacy Policy.*

Reviewer #1: No

________________________________________

We thank the reviewers for their time and expertise.

Regards,

Dr. Brenda L. Coleman, PhD

Clinical Scientist, Sinai Health, Toronto

Assistant Professor, Dalla Lana School of Public Health, University of Toronto

Sinai Health System

600 University Ave

Toronto, ON, Canada M5G 1X5

(C) 647-267-2413

[b.coleman@utoronto.ca](mailto:b.coleman@utoronto.ca)
