# Supplementary material for: Changes in emotional distress among Ontario education workers during the COVID-19 pandemic: 2021–2023
Source: PLoS One. 2025 Aug 18;20(8):e0330442. doi: 10.1371/journal.pone.0330442 (PMC12360544; doi:10.1371/journal.pone.0330442)
Supplement: Attachment — Submitted filename: Response to Reviewers 2024-04-24.docx [file pone.0330442.s004.docx]

April 24, 2025

**RE**: PONE-D-24-47690R1

Changes in emotional distress among Ontario education workers during the COVID-19 pandemic: 2021-2023, PLOS ONE submission

**Response to Reviewers**

We thank the reviewers for their comprehensive reviews. Our responses are captured under each comment. Please note that when lines are referenced, we are referring to the manuscript that highlights changes to the original version, the file labelled “Revised Manuscript with Track Changes”.

**Editor Comments**

*The manuscript requires minor revisions before proceeding further. The reviewer has provided the following comment: "The work-related stress during the COVID-19 pandemic was common among all types of workers across the globe. This paper focuses only on education sector workers. While this cohort study is interesting and useful, it could be more meaningful if the authors include literature on work-related stress among workers in other sectors, particularly the healthcare sector." Addressing this point will enhance the comprehensiveness of the study.*

Literature regarding work-related stress among workers in other sectors has been added to the discussion. Please see lines 321-341 in the track changes copy of the resubmitted manuscript.

**Reviewers' comments:**

Reviewer's Responses to Questions: Comments to the Author

1. If the authors have adequately addressed your comments raised in a previous round of review and you feel that this manuscript is now acceptable for publication, you may indicate that here to bypass the “Comments to the Author” section, enter your conflict of interest statement in the “Confidential to Editor” section, and submit your "Accept" recommendation.

*Reviewer #1: All comments have been addressed*

*Reviewer #2: All comments have been addressed*

________________________________________

2*. Is the manuscript technically sound, and do the data support the conclusions?*

The manuscript must describe a technically sound piece of scientific research with data that supports the conclusions. Experiments must have been conducted rigorously, with appropriate controls, replication, and sample sizes. The conclusions must be drawn appropriately based on the data presented.

*Reviewer #1: Yes*

***Reviewer #2: Partly***

Additional information regarding the impact of the COVID-19 pandemic on workers in other sectors has been added. Please see lines 321-341 in the track changes copy.

________________________________________

*3. Has the statistical analysis been performed appropriately and rigorously?*

*Reviewer #1: Yes*

***Reviewer #2: No***

The Reviewer has not provided any details regarding what aspect of the statistical analysis was not performed appropriately. As no reviewer comments were submitted regarding this issue either in the first or second review, no changes have been made to the manuscript.

________________________________________

*4. Have the authors made all data underlying the findings in their manuscript fully available?*

The PLOS Data policy requires authors to make all data underlying the findings described in their manuscript fully available without restriction, with rare exception (please refer to the Data Availability Statement in the manuscript PDF file). The data should be provided as part of the manuscript or its supporting information or deposited to a public repository. For example, in addition to summary statistics, the data points behind means, medians and variance measures should be available. If there are restrictions on publicly sharing data—e.g. participant privacy or use of data from a third party—those must be specified.

***Reviewer #1: No***

*Reviewer #2: Yes*

The authors believe that his issue has already been addressed.

________________________________________

*5. Is the manuscript presented in an intelligible fashion and written in standard English?*

PLOS ONE does not copyedit accepted manuscripts, so the language in submitted articles must be clear, correct, and unambiguous. Any typographical or grammatical errors should be corrected at revision, so please note any specific errors here.

*Reviewer #1: Yes*

*Reviewer #2: Yes*

________________________________________

*6. Review Comments to the Author*

Please use the space provided to explain your answers to the questions above. You may also include additional comments for the author, including concerns about dual publication, research ethics, or publication ethics. (Please upload your review as an attachment if it exceeds 20,000 characters)

*Reviewer #1: (No Response)*

***Reviewer #2: The work-related stress during COVID-19 pandemic was common among all types of workers across the globe. This paper talks only about the education sector workers. Though this cohort study is interesting and useful, it could be more meaningful if the authors add some literature reviews about the other sector workers particularly health sector workers****.*

Additional information regarding the impact of the COVID-19 pandemic on workers in other sectors has been added. Please see lines 321-341 in the track changes copy.

________________________________________

7. *PLOS authors have the option to publish the peer review history of their article* (what does this mean?). If published, this will include your full peer review and any attached files. If you choose “no”, your identity will remain anonymous but your review may still be made public.

Do you want your identity to be public for this peer review? For information about this choice, including consent withdrawal, please see our Privacy Policy.

*Reviewer #1: No*

*Reviewer #2: Yes: Dr. Jayanta Kuar Basu*

________________________________________

We thank the reviewers for their time and expertise.

If there are any further questions regarding this manuscript, please don’t hesitate to be in touch.

Regards,

Dr. Brenda L. Coleman, PhD

Clinical Scientist, Sinai Health, Toronto

Assistant Professor, Dalla Lana School of Public Health, University of Toronto

Sinai Health System

600 University Ave

Toronto, ON, Canada M5G 1X5

(C) 647-267-2413

[b.coleman@utoronto.ca](mailto:b.coleman@utoronto.ca)
