# Supplementary material for: Changes in emotional distress among Ontario education workers during the COVID-19 pandemic: 2021–2023
Source: PLoS One. 2025 Aug 18;20(8):e0330442. doi: 10.1371/journal.pone.0330442 (PMC12360544; doi:10.1371/journal.pone.0330442)
Supplement: Attachment — Submitted filename: Response to Reviewers 2025_07_24.docx [file pone.0330442.s005.docx]

July 26, 2025

RE: PONE-D-24-47690R1

Changes in emotional distress among Ontario education workers during the COVID-19 pandemic: 2021-2023, PLOS ONE submission

Response to Reviewers

We thank the reviewers for their ongoing support of this work. Our responses are captured under each comment.

If there are any further questions regarding this manuscript, please don’t hesitate to be in touch.

Regards,

Dr. Brenda L. Coleman, PhD

Clinical Scientist, Sinai Health, Toronto

Assistant Professor, Dalla Lana School of Public Health, University of Toronto

Sinai Health System

600 University Ave

Toronto, ON, Canada M5G 1X5

(C) 647-267-2413

b.coleman@utoronto.ca

**1. Deepen the Interpretive Analysis in the Discussion Section**

Location: In the Discussion section, as a new paragraph following the comparison with healthcare and other essential workers (i.e., after line 341 in the track-changes manuscript).

Rationale: To move beyond reporting the comparison and interpret why educators might have experienced higher levels of distress, thereby strengthening the manuscript’s analytical contribution.

Current Text (end of the paragraph):

"...lower than what was found in the current study (2021: 54.7%; 2023: 32.5%) but, in both cases, much higher than what is expected in the general population [44]."

Proposed Text (with new paragraph added):

"...lower than what was found in the current study (2021: 54.7%; 2023: 32.5%) but, in both cases, much higher than what is expected in the general population [44].

The finding that education workers, at times, reported higher levels of distress than even frontline healthcare providers warrants further consideration. This discrepancy may be attributable to several factors unique to the education sector. Unlike healthcare professionals, who are trained for crisis response, educators were thrust into a public health crisis with little to no specific preparation, while simultaneously managing the pedagogical, emotional, and safety needs of children. Furthermore, the intense public and political scrutiny surrounding school closures and reopenings placed educators at the center of a contentious societal debate. This confluence of responsibilities, acting as de facto frontline workers while maintaining educational continuity and navigating significant public pressure, likely created a unique and potent combination of stressors not experienced in the same way by other worker groups."

**Submitted text (lines ):**

The finding, that education workers may have, at times, experienced higher levels of distress than healthcare providers warrants further consideration. A qualitative study conducted in the fall of 2020 among Ontario education workers revealed that teachers were challenged to quickly change their work environment to provide their services using new technologies with limited training opportunities and resources, resulting in increased reports of stress {Serrano, 2023 #144}. When in-person education resumed, teachers were concerned about having to work in an environment with less than ideal measures to reduce the spread of disease {Coleman, 2024 #145}. Furthermore, the intense public and political scrutiny surrounding school closures and re-openings placed educators at the center of a contentious societal debate {Lau, 2022 #143}. These likely created a unique and potent combination of stressors not experienced in the same way by other worker groups.

2**. Strengthen the Policy Implications in the Conclusion Sub-section**

Location: At the end of the "Conclusions" section, following the final sentence (after line 409).

Rationale: To provide a clear, actionable takeaway that highlights the practical and policy relevance of the research findings.

Current Text (final sentence):

"Further research into best practices in early distress identification and remediation is warranted to ensure future pandemics are met with education systems and workers who can continue to provide an exceptional learning experience ."

Proposed Text (with new sentences added):

"Further research into best practices in early distress identification and remediation is warranted to ensure future pandemics are met with education systems and workers who can continue to provide an exceptional learning experience .

Therefore, these findings underscore the need for education authorities to move beyond reactive measures and develop proactive, institutional-level mental health frameworks. Such frameworks should include targeted support for high-risk demographics and provide evidence-based strategies to manage workload and mitigate distress during future public health crises or other systemic disruptions."

**Submitted Text (lines ):**

***Conclusions***

Future pandemics, health emergencies, and other events that lead to education service disruptions need to be met with education systems and workers who can continue to provide an exceptional learning experience. In addition to further longitudinal research into best practices in early distress identification and remediation, a comprehensive, destigmatizing mental health framework targeted to the needs of all education workers that recognises periods of stress intensification as well as high-risk demographic characteristics that can be quickly implemented across school districts and across educational institutions is needed.

**3. Enhance the Link Between Data (Figure 1) and Interpretation in the Discussion Section**

Location: In the Discussion section, within the paragraph discussing the effect of summer vacations (starts around line 345).

Rationale: To explicitly connect the striking visual data from Figure 1 to the interpretation, reinforcing the argument about the impact of acute environmental factors.

Current Text:

"In the current study, distress levels were lower during Ontario education worker vacation periods (summers). This corroborates previous research that reported a strong, but limited, positive effect of vacations on mood [46] ."

Proposed Text (with new sentence integrated):

"In the current study, distress levels were lower during Ontario education worker vacation periods (summers).

The pronounced fluctuations observed in Figure 1, particularly the sharp decrease in distress during summer and the subsequent spike upon returning to the workplace, highlight the significant and immediate impact of work-related environmental factors. This corroborates previous research that reported a strong, but limited, positive effect of vacations on mood [46] ."

**Submitted text (lines ):**

The marked fluctuations observed in Figure 1, particularly the sharp decrease in distress during the summer and the subsequent spike upon returning to the workplace, highlight the significant and immediate impact of work-related environmental factors on emotional well-being. We thank the reviewers for their time and expertise.
